# The Preparations and Water Vapor Barrier Properties of Polyimide Films Containing Amide Moieties

**DOI:** 10.3390/polym9120677

**Published:** 2017-12-05

**Authors:** Kai Zhang, Qiaoxi Yu, Longji Zhu, Siwei Liu, Zhenguo Chi, Xudong Chen, Yi Zhang, Jiarui Xu

**Affiliations:** PCFM Lab, GD HPPC Lab, Guangdong Engineering Technology Research Centre for High-performance Organic and Polymer Photoelectric Functional Films, State Key Laboratory of Optoelectronic Materials and Technologies, School of Chemistry, Sun Yat-sen University, Guangzhou 510275, China; zhangk27@mail2.sysu.edu.cn (K.Z.); yuqx3@mail2.sysu.edu.cn (Q.Y.); zhulj3@mail2.sysu.edu.cn (L.Z.); liusiw@mail.sysu.edu.cn (S.L.); chizhg@mail.sysu.edu.cn (Z.C.); cescxd@mail.sysu.edu.cn (X.C.); xjr@mail.sysu.edu.cn (J.X.)

**Keywords:** polyimides, water vapor barrier property, amide moieties, “locking water” effect

## Abstract

Flexible displays are a systematic revolution in the field of display, in which high-performance and high-barrier polymer substrates are considered to be one of the most important key materials. In this work, high water vapor barrier polyimides containing amide moieties were synthesized via the ternary polymerization of 4,4′-diaminobenzailide (DABA), 4,4′-diaminodipheny ether (ODA), and 3,3′,4,4′-biphenyl-tetracarboxylic acid dianhydride (BPDA) followed by thermal imidization. The relationship between the content of amide moieties and the water vapor barrier property of the prepared polyimides was studied by means of density test, water absorbing test, water contact angle test, water vapor permeation test, fourier transform infrared spectroscopy (FT-IR), thermogravimetric analysis (TGA), thermogravimetry coupled with fourier transform infrared spectrometry (TG-FTIR), wide-angle X-ray diffraction analysis (WXRD), mechanical performance test, etc. The results show that the introduction of amide groups into polyimide (PI) main chains can improve the water vapor barrier properties of the polyimides effectively. The water vapor transmission rate (WVTR) of the polyimide films can be improved from 8.2365 g·(m^2^·24 h)^−1^ to 0.8670 g·(m^2^·24 h)^−1^ with the increasing content of amide moieties.

## 1. Introduction

**Figure 1:**
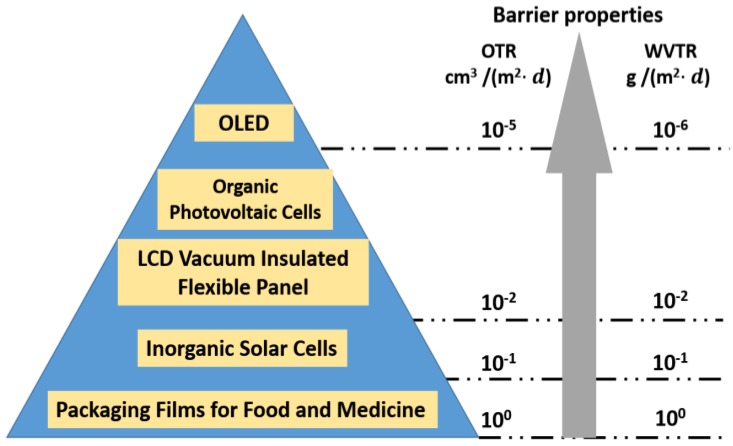
Requirements of products on the barrier performance of barrier materials. OLED: organic light-emitting diode; LCD: liquid-crystal display; WVTR: water vapor transmission rate; OTR: oxygen transmission rate.

The leading development trend of modern electronic products is miniaturization, integration, and portability [1,2]. Among them, flexible display is one of the most compelling systematic revolution technologies. High-performance polymer-based flexible substrates—especially high-barrier polymer substrates—are considered to be one of the most important key materials. They play important roles in the environmental protection and mechanical support for the devices [3,4,5,6,7,8,9,10], and are one of the main factors affecting the lifetime of devices [11,12,13,14,15,16]. The requirements of barrier properties of materials used in different fields are shown in Figure 1. Traditional polymer-based barrier materials are low-cost, good flexibility, light-weight, impact resistant, and require simple processing, and are widely used in the field of electronic encapsulation [17,18,19,20,21,22,23,24,25]. Among the traditional polymer-based substrates (Table 1), polyimides are widely used as electronic packaging and electrical insulation materials in the field of aerospace industries and microelectronics [26,27,28]. They are considered to be the most promising candidates for the flexible substrates of flexible displays because they are thermally stable under high processing temperature. However, the existence of free volume in polymer materials makes them essentially permeable [29,30,31,32,33,34,35,36], thus limiting their applications as barrier materials in the field of flexible displays. To address this, how to effectively reduce the free volume of the polymer and decrease the diffusion rate of water molecules in the polymer become key scientific questions in the field. 

In this work, polyimides containing amide moieties were designed and prepared by solution copolymerization of 4,4′-diamino benzanilide (DABA), 4,4′-diaminodiphenyl ether (ODA) and 3,3′,4,4′-biphenyltetracarboxylic dianhydride (BPDA). Since the amide moieties contain both carbonyl and secondary amine groups, it is easy for them to form hydrogen bonds with hydroxyl groups, amino groups, and other polar groups. On one hand, amide moieties in the polymer backbone may enhance the intermolecular interactions by the formation of hydrogen bonds, which can reduce the free volume of polymers, and thus is beneficial to improve its barrier properties. On the other hand, the formation of hydrogen bond interactions between the amide moieties and the water molecules would contribute to reducing the diffusion rate of water vapor molecules in polymer materials, which also helps to enhance the water vapor barrier properties of the materials [38,39,40]. To investigate these interactional mechanisms, Fourier transform infrared spectroscopy (FT-IR), thermogravimetric analysis (TGA), and thermogravimetry coupled with fourier transform infrared spectrometry (TG-FTIR) were used to characterize the hydrogen bond interactions between the polymer segments and water molecules, and the “locked-water effect” of the amide moieties was proposed. FT-IR, TGA, TG-FTIR, wide-angle X-ray diffraction analysis (WXRD), water absorbing test, water contact angle test, mechanical performance test, and water vapor permeation test were also used to characterize the structure and properties of the polyimides.

## 2. Materials and Methods

### 2.1. Materials

4,4′-Diamino benzanilide (DABA) and 4,4′-diaminodiphenyl ether (ODA) purchased from Alfa-Aesar (Shanghai, China) were used as-received. 3,3′,4,4′-Biphenyltetracarboxylic dianhydride (BPDA) purchased from Shanghai GuChuang New Chemical Materials Co., Ltd. (shanghai, China) was used as-received. *N*,*N*-Dimethylformamide (DMF) was provided by Guangzhou Chemical Reagent Factory (Guangzhou, China).

### 2.2. Synthesis of Poly(Amic Acid) Copolymers and Preparations of PI Films

A representative example of synthesizing poly(amic acid) (PAA) copolymer was described as follows, where the mole ratio of DABA to ODA is 5:5, and the sample is marked as 5O5DPAA. Monomer-grade diamines, DABA (2.2985 g, 10.1 mmol) and ODA (2.0251 g, 10.1 mmol), were added into a dry argon-flushed round-bottom flask. After the diamines had completely dissolved in anhydrous DMF (50 mL) by mechanical stirring at 25 °C, dianhydride monomer BPDA (6.0704 g, 20.06 mmol) was added into the solution in three batches. After the reaction had proceeded for 6–7 h, viscous 5O5DPAA solution was obtained with a solid content of 18 wt %. The PAA was subsequently coated uniformly on a clean and dry glass plate with a controlled film thickness, and then underwent a thermal imidization process in a vacuum oven with temperature program of 100 °C (1 h)/200 °C (1 h)/300 °C (1 h) to produce the final polyimide 5O5DPI. The polyimide film was removed from the glass substrate after the oven cooled to room temperature. Other polyimide films with different diamine ratios (ODA:DABA = 10:0, 7:3, 3:7, 0:10) were prepared in the same way, and were marked as OPI, 7O3DPI, 3O7DPI, and DPI.

### 2.3. Methods

FT-IR spectra were recorded on a Fourier transform infrared spectrometer (Nexus 670, Nicolet, WI, USA) from 4000 cm^−1^ to 400 cm^−1^ using attenuated total reflection (ATR) mode to characterize the chemical structure of the polyimide films. WXRD experiments were performed on a SmartLab LIFM-X (Rigaku, Tokyo, Japan) with a copper target tube and a two-dimensional detector. The X-ray beam was monochromatized to Cu Kα radiation (λ = 1.54 Å) and the X-ray generator was operated at 40 kV and 30 mA. The 2θ ranged from 5° to 40°. The drainage method was used to measure the density of the dry PI films at room temperature. Before the test, the polyimide films were placed in an oven and heated to 300 °C with a 24 h isotherm to remove residual moisture from the films. An optical contact angle measuring instrument (DSA100, Kruss, Hamburg, Germany) was used to detect the hydrophilicity of the polyimide films, and the volume of water drop was 2 μL. The water absorption test method was as follows: dry films were weighed by electronic analytical balance to get the mass *M*_1_, and then the dry films were immersed in deionized water for 168 h to make the films saturated with water. The surface was wiped and the films were weighed to get the mass *M*_2_; then, the water absorption of the film could be obtained by (*M*_2_ − *M*_1_)/*M*_1_ × 100%. Thermal properties were determined through thermogravimetric analysis (TG-Q50, TA Instruments, New Castle, DE, USA). TGA was carried out from 50 °C to 900 °C at a heating rate of 20 °C/min under N_2_. A TG-FTIR interconnection instrument (TG-209, Netzsch, Freistaat Bayern, Germany/Vector-22, Brucker, Billerica, Germany) was used to identify the composition of volatile at a heating rate of 20 °C/min under N_2_. The mechanical properties were determined on a CMT6103 mechanical measuring instrument (SANS, Shenzhen, China) at a drawing rate of 10 mm/min, the samples were 10 mm in length and 100 mm in width. The water vapor transmission rates (WVTRs) were measured using a permeation test system (W3/330, Labthink, Ji’nan, China) at 37.8 °C and 85% relative humidity (RH).

## 3. Results and Discussion

### 3.1. Synthesis and Characteristics of the Polyimide Films

**Scheme 1:**
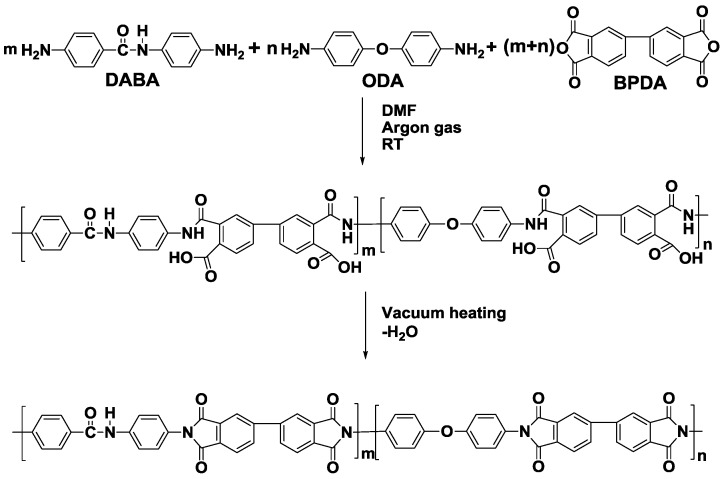
Scheme of the synthesis of polyimide containing amide moieties. BPDA: 3,3′,4,4′-biphenyltetracarboxylic dianhydride; DABA: 4,4′-diamino benzanilide; ODA: 4,4′-diaminodiphenyl ether. DMF: *N,N*-Dimethylformamide; RT: room temperature.

**Figure 2:**
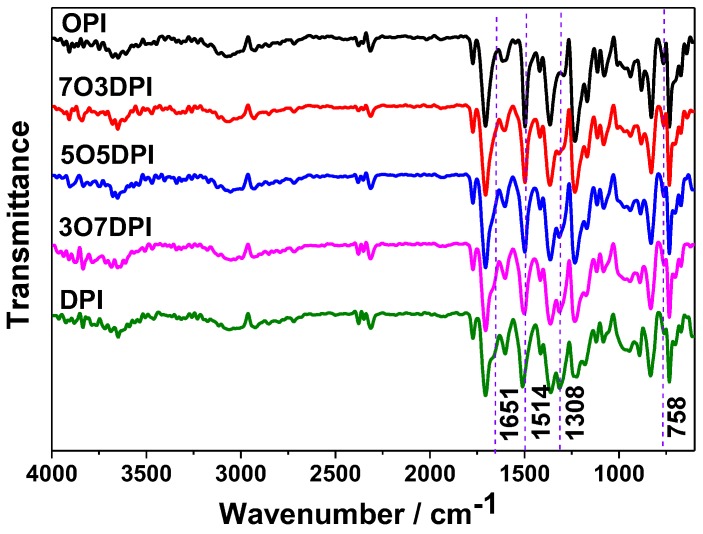
Fourier transform infrared (FT-IR) spectra of the polyimide films. OPI refers to a polyimide film where ODA:DABA = 10:0; 7O3DPI a film where ODA:DABA = 7:3; 5O5DPI a film where ODA:DABA = 5:5; 3O7DPI a film where ODA:DABA = 3:7; DPI a film where ODA:DABA = 0:10.

The amide-containing polyimides were synthesized using a two-step method by the copolymerization of ODA and BPDA with an amide-containing diamine DABA, as shown in Scheme 1. The poly(amic acid)s were synthesized by gradually adding the dianhydride monomer into the anhydrous DMF solution of diamines, and stirred for 6–7 h at 25 °C. Thermal imidization procedures were chosen to form polyimides. The chemical structure of the polyimide films was investigated by FT-IR, and the results are shown in Figure 2. The polyimide films exhibited characteristic absorption peaks of imide ring at around 1779 cm^−1^ (C=O asymmetrical stretching vibration), 1725 cm^−1^ (C=O symmetrical stretching vibration), 1380 cm^−1^ (C–N stretching vibration), and 758 cm^−1^ (imide ring deformation). These demonstrate that all of the poly(amic acid)s were fully converted into polyimides by the thermal imidization process. When DABA was added, the polyimide films exhibited characteristic absorption peaks at 1651 cm^−1^ (“Amide I”, C=O stretching vibration in amide), 1514 cm^−1^ (“Amide II”, C–N–H bending vibration), and 1308 cm^−1^ (“Amide III”, the mix of C–N stretching vibration and N–H bending vibration). This indicated that the synthesized polyimide had characteristics of both imide and amide groups. Additionally, the absorption peak intensity of the peaks at 1651 cm^−1^ and 1308 cm^−1^ increased with the increase of DABA content.

### 3.2. Aggregation Structures and Density of the Polyimide Films

**Figure 3:**
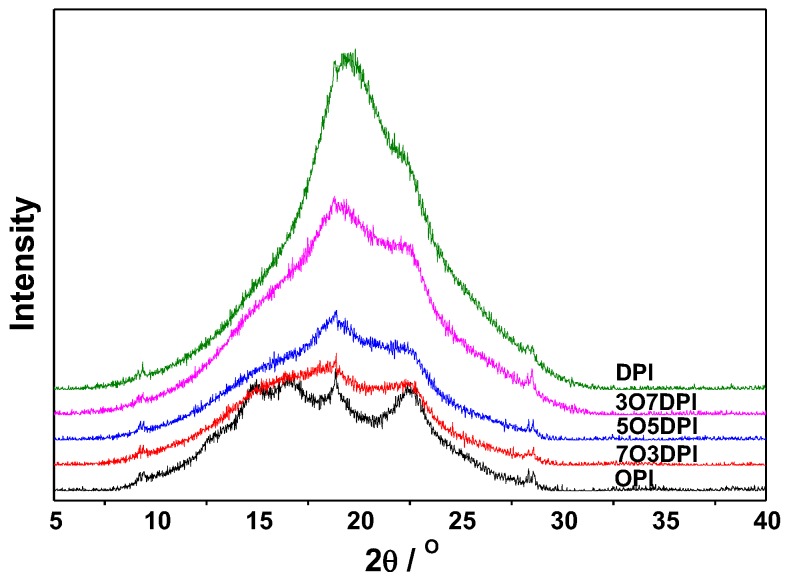
Wide-angle X-ray diffraction (WXRD) spectrum of the polyimide films.

The aggregation state of the polyimide films was characterized by WXRD with graphite monochromatized Cu K*α* radiation, 2θ ranging from 0° to 40°, and the results are shown in Figure 3. The X-ray diffraction curves of the polyimide films express a set of wider diffraction peaks in the range of 10° to 33°. With the increase in DABA content, the intensity of the diffraction peaks increased and the shape become sharper, which meant that the order of the polyimide macro-molecular chain arrangement increased. The reason for this may be due to the existence of the amide-group in the polymer main chain, which is conducive to the formation of inter-molecular hydrogen bond interactions, thus enhancing the inter-molecular interactions to form more ordered and compact macromolecular chain arrangements. 

**Figure 4:**
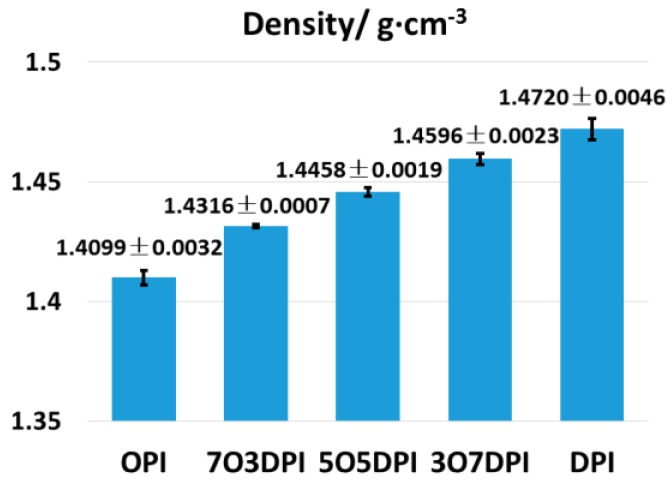
Density data of the polyimide films.

The density of the polyimide films was measured by the drainage method, and the results are shown in Figure 4. The density of the polyimide without DABA moiety (OPI) was 1.4099 ± 0.0032 g/cm^3^. With the increase of DABA content, the density of polyimide film increased, from 1.4316 ± 0.0007 g/cm^3^ (7O3DPI) to 1.4720 ± 0.0046 g/cm^3^ (DPI). The density increased by 16.9% when the diamine ODA was completely replaced by DABA. Combined with the WXRD results, it is obvious that the increase in density may be due to the closest packing of the macromolecules caused by the inter-molecular hydrogen bonding interaction. 

### 3.3. Surface Hydrophilicity and Water Absorption Properties of the Polyimide Films

**Figure 5:**
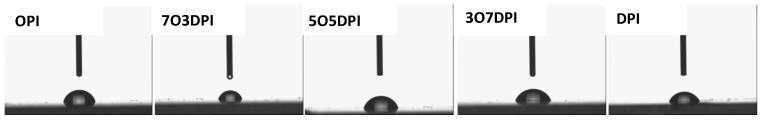
Images of water contact angle test of polyimide films.

The introduction of the amide moiety into the polyimide backbone increases the molecular polarity of the prepared polyimides, which will have an effect on the surface polarity and water absorption capacity of the polymer. The hydrophilicity of the polyimide films was measured by an optical contact angle measuring instrument. The results in Figure 5 and Table 2 show that all the polyimide films exhibited certain hydrophilic properties. The surface water contact angle obviously decreased with the increase of the DABA content, ranging from 79.4° (OPI) to 64.7° (DPI). This means that the increase of the polar amide groups in the polyimide backbone resulted in the hydrophilic enhancement of the surface.

Before the water absorption test, the polyimide films were placed in a vacuum oven at 300 °C for 24 h to ensure that the films were completely dry. The dried films were weighed by an electronic analytical balance to get the mass *M*_1_. Then, the dry films were immersed in deionized water for 168 h to make sure the polyimide films were fully absorbent and saturated with water. Then, the film surface was wiped and the film was weighed to get the mass *M*_2_. The water absorption of the film can be calculated by the equation (*M*_2_ − *M*_1_)/*M*_1_ × 100%. The results are shown in Table 2. As can be seen, the water absorption of polyimide films increased dramatically with the increase of the DABA content, from 0.7998% (OPI) to 3.6327% (DPI). As it is known that the amide groups in the polymer main chain can easily form hydrogen bonds with water molecules, so the water absorption capacities of the polymers enhanced. 

### 3.4. Mechanical Properties of the Polyimide Films

The mechanical properties of the polyimide films were tested on an electronic universal testing machine. The results are shown in Table 3. All polyimide films exhibit excellent mechanical properties. The tensile strength of OPI film is 136.8 ± 4.7 MPa, the elastic modulus is 3.20 ± 0.51 GPa, and the elongation at break is 14.4 ± 2.3%. With the increase of the DABA fraction in the polymer backbone, the tensile strength and elastic modulus increased, and when all the ODA was replaced by DABA, they reached 174.3 ± 0.6 MPa and 4.08 ± 0.29 GPa, respectively. However, the elongation at break decreased from 14.4% to 9.8%. Obviously, although the introduction of rigid DABA components will have a certain impact on the flexibility of the materials, they are beneficial to improve their mechanical strength. 

Hedenqvist proved that water can affect the structure of amorphous proteins by forming different complex structures at different relative humidities, and demonstrated how this affects the mechanical behavior [41]. To understand the influence of the existence of water in the polyimide film on the properties of the materials, we also studied the mechanical properties of the films after soaking in deionized water for 168 h. The results in Table 4 indicate that the water molecules remaining in the polyimide films had little effect on the mechanical properties of the materials.

### 3.5. Water Vapor Barrier Properties of the Polyimide Films

WVTR were measured using a Permeation Test System at 37.8 °C and 85% RH, and the results are listed in Table 5. As can be seen, the transmission coefficient and WVTR of OPI is 1.261 × 10^−12^ g·cm·(cm^2^·s·Pa)^−1^ and 8.2365 g·(m^2^·24 h)^−1^, respectively. To our surprise, the WVTR of the polyimide films decreased from 8.2362 g·(m^2^·24 h)^−1^ (OPI, polyimide without DABA segments) to 0.8670 g·(m^2^·24 h)^−1^ (DPI, polyimide without ODA segments) with the increase of DABA content. The water vapor barrier properties of the amide-containing polyimide films were improved obviously. Combined with the above experimental results, it suggests that with the increase in the polarity of the polyimide, stronger hydrophilicity of the polymer is more conducive to improving its water vapor barrier properties.

### 3.6. Mechanism of Water Vapor Barrier Properties of the Polyimides

The hydration water in a solid medium can be categorized into two types: free water and bound water. The free water almost does not interact with the contact medium, while the bound water is an extremely thin layer of water surrounding the medium surfaces, which has a strong attraction between the surfaces and the water molecules, and is much less mobile than the rest of the water in the medium. According to the distance and the interaction strength between the water molecules and the medium, the bound water can be divided into loosely-bound water and tightly-bound water. At the same time, it is also known that in the polymer, the permeation process of gas molecules can be divided into adsorption, diffusion, and desorption steps. Each step is closely related to the interaction between the gas molecules and materials, and has an important effect on the barrier properties of the material. So, this work shows that the nature of the water–polyimide coupled interactions are essential to the water vapor barrier properties of the polyimide. 

**Figure 6:**
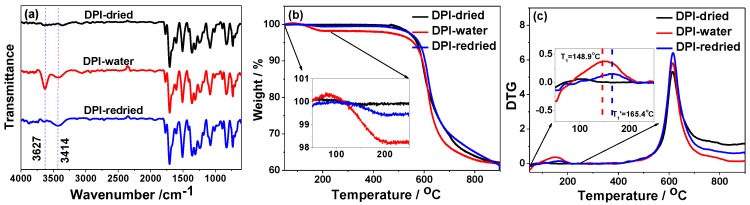
FT-IR spectra and thermogravimetric analysis (TGA) curves of the DPI films under different conditions. (**a**) FT-IR spectra; (**b**) TGA curves; (**c**) Derivative thermogravimetric analysis (DTG) curves.

FT-IR and TGA tests were performed on the DPI films obtained under different treatment conditions. DPI-dried film was obtained by drying the original polyimide film at 300 °C for 24 h. DPI-water film was obtained by immersing the DPI-dried film into deionized water for 168 h to make sure the polyimide films were saturated with water. Then, the DPI-water film was dried at 100 °C for 24 h to get a DPI-redried film. The results are shown in Figure 6a–c. For the DPI-dried film, there is a very small absorption peak at 3300–3700 cm^−1^ (Figure 6a), and almost no weight was lost in the temperature range of before 450 °C as the TGA curves shown in Figure 6b; hence, the absorption peaks should come from the N–H stretching vibration of the amide bond in the backbone. The FT-IR spectrum of the DPI-water film exhibited strong absorption peaks at 3627 cm^−1^ and 3414 cm^−1^, which is the free hydroxyl stretching vibration and the hydrogen-bonded hydroxyl stretching vibration, respectively, and indicates that both free water and hydrogen-bonded water existed in the DPI-water film. Additionally, as shown in Figure 6b,c, all of these water molecules can be completely removed before 215 °C, with a weight loss of about 1.76%. For the DPI-redried film, only the characteristic absorption peak of hydrogen-bonded hydroxyl stretching vibration (3414 cm^−1^) was observed. The content of this part of water is about 0.65%, which should be the tightly-bound water and can be completely removed before 215 °C (Figure 6b,c). These results indicate that the free water molecules can be removed around 100 °C by evaporation, while the hydrogen-bonded water molecules still existed in the polymer film. This means that after heat treatment at 100 °C in the vacuum oven, the hydrogen-bonded water remained in the polyimide film. These water molecules are “locked” by forming a strong hydrogen bond interaction with the amide group of the polyimide, and are likely to be removed at a temperature higher than 100 °C [42,43,44,45,46].

**Figure 7:**
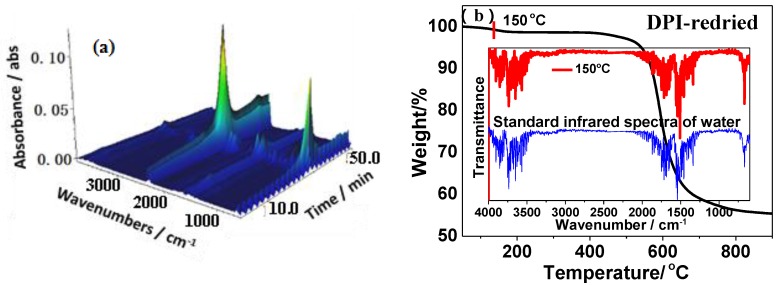
TG-FTIR curves of the DPI-redried film. (**a**) TG-FTIR curves; (**b**) FT-IR spectrum of the volatiles at 150 °C.

In order to look further into the weight loss process of the polyimide film in the temperature range of 50–215 °C, the structure of the volatiles was characterized by TG-FTIR. Taking DPI-redried as an example, the results are shown in Figure 7a,b. From the overall FT-IR mapping of the volatile products during the TGA test (Figure 7a), it can be seen that there are two main decomposition processes, which correspond to the TGA results. The FT-IR spectrum of the volatile products at 150 °C is shown in Figure 7b. The results are consistent with the standard infrared spectrum of gaseous water (inserted picture in Figure 7b). It is shown that the volatile product in this temperature range is the water component. Usually, water molecules should evaporate at about 100 °C. However, water molecules that interacted with amide groups by hydrogen bonds are more difficult to evaporate; that is, these “locked” water molecules require more energy to escape from the film [47,48], so the evaporation temperature of these water molecules is much higher than 100 °C and can only be completely removed at about 215 °C.

**Figure 8:**
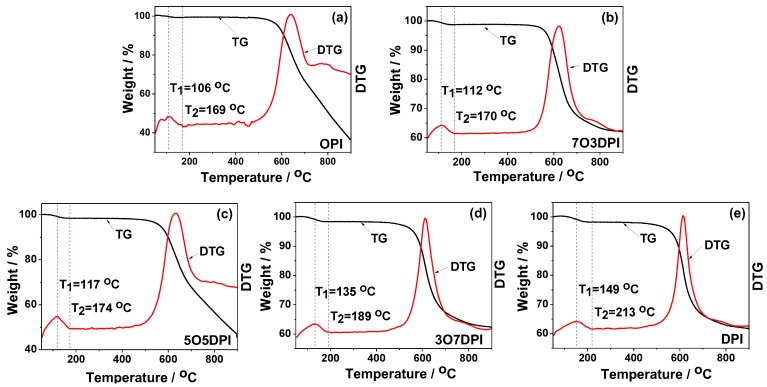
TG and DTG curves of the saturated water-absorbing polyimide films. (**a**) OPI; (**b**) 7O3DPI; (**c**) 5O5DPI; (**d**) 3O7DPI; (**e**) DPI.

To investigate the influence of DABA content on the dehydration temperature of the hydration water in the amide-containing polyimide films, the thermogravimetric behaviors of the saturated water-absorbing polyimide films were studied by TGA, and the results are shown in Figure 8a–e. There are two mass loss processes on the TGA curves. The high-temperature process (above 500 °C) corresponds to the thermal decomposition of the polyimide backbone. The mass loss process in the range of 50–400 °C is closely related to the removal of water molecules in the films according to the TG-FTIR results.

On the DTG curves, the peak value of the first weight loss peak is labeled as *T*_1_, which is the temperature of the highest mass lost rate; and *T***_2_** refers to the temperature when the weight loss ended. For the OPI-water sample, as shown in Figure 7a, the hydrate water was fully removed before 169 °C (*T*_2_). When 30% DABA was introduced into the polyimide backbone (7O3DPI), *T*_1_ was 112 °C, increased by 6 °C, as compared with OPI; while *T*_2_ remains almost the same. Both *T*_1_ and *T*_2_ were obviously increased with the increase of the DABA content, and finally reached 149 °C and 213 °C when all the ODA was replaced by DABA (sample DPI-water), increased by 43 °C and 44 °C, respectively. At the same time, with the increase of DABA content, the weight loss of water in this system was increased, which is 0.54%, 1.15%, 1.38%, 1.60%, and 1.76%, respectively. These results indicate that the hydrogen-bond interactions between water molecules and the amide moieties could “lock” water molecules in the films, and the more amide moieties [49], the more water molecules are locked; meanwhile, the water removal temperature was increased with increasing DABA content.

**Figure 9:**
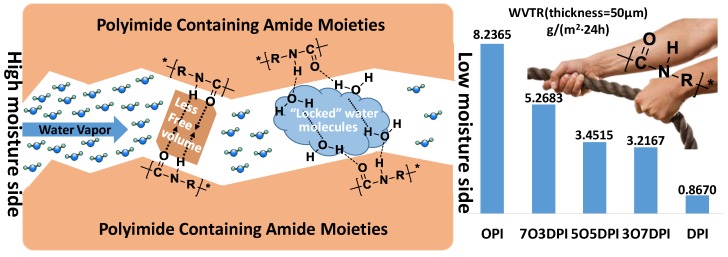
The barrier mechanism of the amide-containing polyimide films.

Based on the above results, the barrier mechanism of the amide-containing polyimides can be proposed, as shown in Figure 9. On one hand, by forming the strong hydrogen bonding intermolecular interaction, the amide groups in the polyimide backbone can improve the orderly arrangement of the polyimide macromolecules, which is beneficial to increase the tight stacking of the molecular chain, thus increasing the density of the polyimide film. On the other hand, the amide groups can interact with water molecules through strong hydrogen bond, so that the penetrated water molecules can be “locked” in the films, and their diffusion in the film would be greatly limited; the “locked” water molecules can also form hydrogen bonds with other water molecules. These “locked” water molecules may form certain “water clusters” in the film, which needs to be further confirmed by some direct experimental methods such as neutron scattering. The “locked” water molecules in the film may further decrease the free volume of the polymer, and thus help to improve the WVTR property of the materials. 

**Figure 10:**
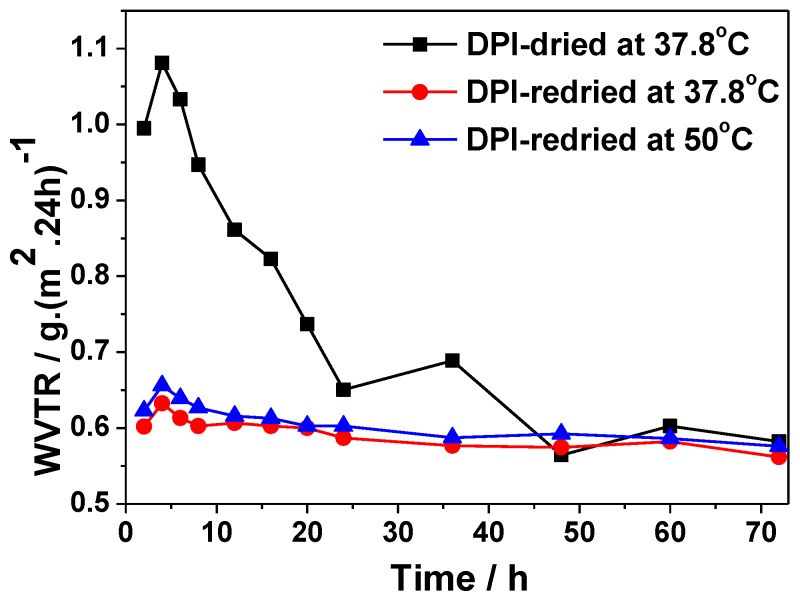
Water barrier property of DPI films (45 μm in thickness, testing at 85% RH for 72 h).

In order to further verify the mechanism proposed above, WVTR measurements were performed on DPI samples (diameter of 1.2 cm and thickness of 45 μm) for 72 h under different pretreatment or test conditions. The DPI-dried film was obtained by drying the polyimide film at 300 °C for 24 h to ensure it was dried completely. WVTR test was carried out at 37.8 °C and 85% humidity, and the results are shown in Figure 10. With the increase of testing time, the WVTR showed a trend of increasing first and then decreasing (black line in Figure 10). The WVTR reached the highest value of 1.083 g·(m^2^·24 h)^−1^ when the testing time was 4 h, and then the WVTR fell steadily, finally stable at around 0.6000 g·(m^2^·24 h)^−1^ after the testing time was greater than 48 h. This phenomenon can be interpreted as follows. At the very beginning, the WVTR is at a relatively high level. As the test progressed, the water vapor molecules continued to penetrate into the interior of the film, and were “locked” in the film by forming strong hydrogen bond interactions with the amide bond on the macromolecular chain. The water molecules entering later may also form hydrogen bond with the “locked” water molecules, filling the voids (also known as free volume) inside the polymer film. The existence of these “locked” water molecules will prevent the further diffusion process of water molecules in the film, thus making the penetration or diffusion of the water molecules increasingly difficult. As a result, the WVTR of the polyimide film is continuously lowered, and the barrier property thereof is improved.

The effect of the tightly-bound water molecules on the water vapor barrier properties of the polyimide films can be further verified by the WVTR test results of the DPI-redried film. The sample was obtained by drying the water-saturated DPI film in a 100 °C vacuum oven, and it has been determined that there was about 0.65% of tightly-bound water in the film (Figure 6). The WVTR testing condition was also at 37.8 °C and 85% humidity, and the results are shown in Figure 10 (red line). Compared with the DPI-dried sample (black line), the barrier property of the DPI-redried sample was more stable during the test and the WVTR values were maintained at a relatively lower level, which is about 0.6000 g·(m^2^·24 h)^−1^—almost the same as that of the DPI-dried sample after 48 h testing. More importantly, such good water vapor barrier properties are stable at a higher temperature, as shown by the blue line in Figure 10, which was measured at 50 °C (the highest testing temperature of the equipment) and 85% RH for 72 h. 

## 4. Conclusions

In summary, an interesting and novel approach was shown to improve the water vapor barrier property of polymer materials by introducing a typical polar group (amide group) into the polymer backbone, and a so-called “locked-water effect” was proposed to illuminate the mechanism. Our results showed that the existence of the amide groups in the polymer main chain played an important role in the WVTR property in two aspects. On the one hand, the formation of inter-molecular hydrogen bond interactions among the amide groups is beneficial to form a more ordered and compact aggregation structure. On the other hand, the amide groups can form strong hydrogen bond interactions with the water vapor molecules, preventing their diffusion by “locking” them in the film, these “locked” water molecules in the film may further decrease the free volume of the polymer, and thus help to improve the WVTR property of the materials. By this means, the WVTR value of the polyimide films can be decreased by an order of magnitude, from 8.2362 g·(m^2^·24 h)^−1^ to 0.8670 g·(m^2^·24 h)^−1^. 

## Figures and Tables

**Table 1:** Water vapor permeation of common commercial barrier polymers [37].

Company	Model	Type	WVTR (50 μm)/g·(m^2^·24 h)^−1^
uPont	Kapton	Polyimide(PI) Film	27
UBE	Upilex R	PI Film	11.2
Sabic Innovative Plastics	Ultem 1000	Polyetherimide (PEI) Film	60
Dow Chemical	Trycite Oriented	Polystyrene(PS) Film	70
Teijin	Mylar	Polyethylene terephthalate(PET) Film	10.6
EMS Chemie	Grivory G21	Nylon	7
Chevron Phillips	Ryton	Polyphenylene sulfide(PPS) Films	6

**Table 2:** Water absorption and water contact angle of the polyimide films.

Sample	*M*_1_/g	*M*_2_/g	Water absorption/%	Water contact angle/°
OPI	0.6001	0.6049	0.7998	79.4
7O3DPI	0.3065	0.3112	1.5334	75.3
5O5DPI	0.2563	0.2618	2.1459	72.7
3O7DPI	0.3072	0.3154	2.6693	68.1
DPI	0.2505	0.2596	3.6327	64.7

**Table 3:** Mechanical properties of the polyimide films.

Sample	Tensile strength/MPa	Elongation at break/%	Elastic modulus/GPa
OPI	136.8 ± 4.7	14.4 ± 2.3	3.20 ± 0.51
7O3DPI	140.2 ± 4.5	12.6 ± 1.3	3.58 ± 0.38
5O5DPI	152.7 ± 4.3	12.5 ± 1.1	3.65 ± 0.49
3O7DPI	165.4 ± 6.4	11.0 ± 2.4	3.66 ± 0.30
DPI	174.3 ± 0.6	9.8 ± 1.6	4.08 ± 0.29

**Table 4:** Mechanical properties of the polyimide films after saturated with water.

Sample	Tensile strength/MPa	Elongation at break/%	Elastic modulus/GPa
OPI-water	134.6 ± 2.5	13.5 ± 2.1	3.07 ± 0.39
5O5DPI-water	155.9 ± 3.9	11.7 ± 1.9	3.70 ± 0.37
DPI-water	176.0 ± 3.5	9.6 ± 1.4	4.27 ± 0.37

**Table 5:** Water barrier properties of polyimide films *.

Sample	Transmission coefficient/g·cm·(cm^2^·s·Pa)^−1^	WVTR/g·(m^2^·24 h)^−1^
OPI	1.261 × 10^−12^	8.2365
7O3DPI	8.063 × 10^−13^	5.2683
5O5DPI	5.282 × 10^−13^	3.4515
3O7DPI	4.923 × 10^−13^	3.2167
DPI	1.327 × 10^−13^	0.8670

* Samples are 50 μm in thickness, testing at 37.8 °C and 85% RH for 18 h.
